# Chewing Matters: Masticatory Function, Oral Microbiota, and Gut Health in the Nutritional Management of Aging

**DOI:** 10.3390/nu17152507

**Published:** 2025-07-30

**Authors:** Monia Lettieri, Alessio Rosa, Fabrizio Spataro, Giovanni Capria, Paolo Barnaba, Marco Gargari, Mirko Martelli

**Affiliations:** 1 Section of Hygiene, Department of Life Sciences and Public Health, Università Cattolica del Sacro Cuore, 00136 Rome, Italy; 2 Department of Clinical Sciences and Translational Medicine, University of Rome Tor Vergata, Via Montpellier 1, 00133 Rome, Italy; 3 Section of Clinical Nutrition and Nutrigenomics, University of Rome Tor Vergata, 00133 Rome, Italy; 4 SSD Centre for Artificial Nutrition, Antonio Cardarelli Regional Hospital, 80131 Campobasso, Italy

**Keywords:** masticatory function, oral microbiota, gut health, aging and nutrition, malnutrition in the elderly

## Abstract

Aging is a multifactorial process that affects various physiological functions, including masticatory performance, which is crucial for oral health and nutritional well-being. Impaired masticatory function, often due to factors such as tooth loss, reduced salivation, or muscle atrophy, can lead to significant nutritional challenges and compromise the overall health of elderly individuals. Recent research has illuminated the interconnectedness of masticatory function, oral microbiota, and gut health, suggesting that altered chewing ability may disrupt oral microbial communities, which in turn affect gastrointestinal health and systemic inflammation. This commentary review provides a comprehensive analysis of the role of masticatory function in aging, exploring its impact on the oral microbiota, gut health, and broader nutritional status. We discuss the potential consequences of impaired mastication, including malnutrition, dysbiosis, and gastrointestinal disorders, and explore possible strategies for improving masticatory function and maintaining a healthy gut microbiome through interventions like dietary modifications, oral care, and rehabilitation. We aim to underscore the importance of integrating masticatory function management into the broader context of aging-related healthcare, promoting holistic, multidisciplinary approaches to support nutritional needs and quality of life in older adults.

## 1. Introduction

Aging is a multifactorial physiological process that leads to significant changes in several bodily functions, including those related to oral and digestive health. As individuals age, there is often a decline in oral health, such as tooth loss, reduced masticatory strength, and decreased salivation, alongside changes in gastrointestinal function, including slowed motility and alterations in the gut microbiota composition [1]. Dysbiosis, an imbalance in the microbial community marked by reduced diversity and the prevalence of pro-inflammatory species, is more commonly observed in elderly individuals compared to younger adults [2]. While the digestive system maintains remarkable resilience with age, comorbidities and the aging process itself can negatively affect nutrient digestion, absorption, and gut integrity [3]. Approximately 18% of people over 65 in Japan have an inadequate nutritional status [4]. In Europe, the prevalence of malnutrition among community-dwelling older adults is estimated to range between 10% and 30%, depending on country and setting [5]. Globally, the World Health Organization reports that up to one in three elderly individuals is at risk of malnutrition, particularly in institutionalized or hospitalized populations.

Mastication is the first stage of digestion and serves multiple essential roles in ensuring effective nutrient utilization. By mechanically breaking down food and mixing it with saliva (which contains digestive enzymes such as amylase), mastication facilitates further digestion and nutrient absorption in the small intestine. Additionally, the act of chewing triggers neurophysiological responses at the central level (cephalic-vagal reflexes) that stimulate the secretion of gastric and pancreatic juices and increase gastrointestinal motility [6]. For example, the simple act of chewing gum post-surgery is used clinically to stimulate bowel transit and promote the recovery of digestive function after surgical procedures [7]. In contrast, dietary habits that limit masticatory stimulation, such as diets composed predominantly of soft or liquid foods, can alter the digestive and microbial balance. Experimental evidence in animal models shows that the prolonged absence of mastication has detrimental effects on the gut ecosystem: mice fed a powdered diet (lacking solid textures) show decreased levels of beneficial short-chain fatty acid (SCFA)-producing bacteria in the cecum, along with mild colonic inflammation and constipation-like symptoms, effects that can be partly reversed by reintroducing adequate masticatory activity [8]. This suggests that food texture and the need for mastication not only affect mechanical digestion but also influence microbial composition and gut health through the diet–microbiota–host interaction.

Given these observations, the hypothesis has been put forward that reduced masticatory function may contribute to both dysbiosis and malnutrition in older adults. Recent evidence supports this hypothesis both biologically and clinically. On the one hand, studies of the oral microbiota have shown that masticatory ability significantly influences the microbial ecosystem in the mouth: elderly individuals with significant masticatory difficulties exhibit decreased salivary microbiota diversity and altered bacterial composition compared to those with normal mastication [9]. In particular, reduced microbial diversity and a relative increase in certain fermentative bacterial genera (such as *Lactobacillus*) in patients with poor mastication suggest oral dysbiosis associated with impaired masticatory ability [10]. Since the oral microbiota acts as a “gateway” to the gastrointestinal tract, such alterations may have repercussions for the gut microbiota, for instance, through the continuous swallowing of oral bacteria and reduced saliva production (which normally helps control pathogens). On the other hand, impaired mastication affects the dietary choices of older individuals, often leading them to select easier-to-chew foods that are nutritionally poorer. Numerous nutritional studies have confirmed that older adults with severe tooth loss or low masticatory efficiency tend to have reduced intake of many nutrient classes: for example, their intake of proteins, fiber, minerals (such as potassium and calcium), and vitamins is significantly lower than that of individuals with functional dentition [11]. Thus, it is not surprising that difficulty chewing is also associated with an increased risk of malnutrition. In a study conducted on over 500 independent elderly individuals, those with poor masticatory ability had lower caloric and nutrient intakes (except for refined carbohydrates), and a higher prevalence of signs of protein-energy malnutrition; this association remained significant after adjusting for confounding variables such as age, cognitive status, tooth number, and comorbidities [12]. In other words, reduced masticatory function is one of the determinants of malnutrition in older adults. This phenomenon is part of a broader condition known as “oral frailty”: a decline in oral function (such as tooth loss, weak mouth muscles, and difficulty chewing and swallowing) that has been linked to an increased risk of disability, sarcopenia, and mortality in older adults [13]. Oral and nutritional frailty often feed into each other, creating a vicious circle in which poor mastication contributes to malnutrition and microbial imbalance, which, in turn, worsens the overall health of frail older adults.

These connections between mastication, microbiota, and nutritional status have led to a growing clinical interest in adopting a more holistic approach to healthy aging. Geriatric dentistry and clinical nutrition are converging in recognizing that “chewing matters”: preserving masticatory function and the balance of the oral–gut microbiota is an integral part of strategies to improve the quality of life and health of older adults. Consequently, there is increasing emphasis on multidisciplinary interventions. On the one hand, maintaining and rehabilitating oral function (through prosthetic dental care, oral hygiene, and masticatory exercises) can lead to nutritional benefits; on the other hand, supporting nutritional status helps protect oral health in a virtuous circle. Recent publications explicitly recommend integrating nutrition professionals into geriatric care plans that involve oral health management: for example, in a study, 65.9% of elderly patients assessed in nutrition clinics were found to be malnourished or at risk of malnutrition, a condition strongly associated with poor oral health, leading the authors to suggest the systematic inclusion of dietitians in the care team to improve the nutritional status of these patients [14]. Similarly, other authors have concluded that the comprehensive nutritional management of older adults should include close collaboration between nutritionists and dentists, addressing both dietary and oral aspects of a patient’s care [15]. In summary, clinical nutrition is shifting toward a more-integrated perspective in which mastication, microbiota, and nutrition are jointly considered: a holistic approach that is essential for supporting healthy aging, with masticatory function recognized as a key determinant of well-being in later life [16]. Despite the growing recognition of these interconnections, the integration of oral function into nutritional care frameworks for aging populations remains inconsistent across clinical practice. The historical separation between dental and nutritional sciences has often led to fragmented interventions, limiting their long-term effectiveness. There is a pressing need to unify these domains under a shared framework of “oral-nutritional health” to address the complex challenges of aging more effectively.

## 2. Materials and Methods

This scoping review is based on a thorough review of the existing scientific literature, particularly focusing on the role of masticatory function, oral microbiota, and gut health in the nutritional management of aging. The approach was primarily qualitative and aimed to synthesize findings from a variety of research fields, including geriatric nutrition, dentistry, microbiology, and gastrointestinal health.

### 2.1. Literature Search

The review was conducted by searching for peer-reviewed articles and clinical studies published in English, primarily between 2018 and 2024, using academic databases such as PubMed, Scopus, and Google Scholar. Keywords used in the search included “masticatory function,” “oral microbiota,” “gut health,” “aging,” “malnutrition,” “nutrition in elderly,” and “oral frailty.” The search focused on studies that investigated the relationship between masticatory dysfunction, oral microbial composition, and gastrointestinal health in the elderly population. Both clinical and experimental studies were included to provide a comprehensive perspective on the topic. Clinical studies encompassed observational cohorts, cross-sectional surveys, and interventional trials primarily involving older adults, assessing parameters such as masticatory efficiency, nutritional intake, and microbiota composition. Experimental studies, particularly those involving animal models, investigated mechanistic links between chewing function, gastrointestinal physiology, and microbial alterations. Together, these two research modalities enabled a more-robust synthesis of evidence, combining real-world clinical insights with controlled experimental data to elucidate cause–effect relationships. ‘Good’ and ‘poor’ masticatory function were defined based on factors such as the number of functional teeth, a subjective assessment scale, and the use of a prosthesis, according to Yamamoto [1] and Matsuo [2]. In this review, masticatory function is categorized into two levels—good and poor—based on clinical criteria reported in population studies. Individuals with good masticatory function are typically characterized by the presence of 20 or more functional teeth, adequate occlusal contact in posterior regions, and/or the effective use of dental prostheses. This group generally reports no significant difficulty in chewing a variety of food textures. Conversely, poor masticatory function is defined by factors such as severe tooth loss, the absence of functional prostheses, reduced occlusal contacts, and self-reported difficulties chewing foods that require moderate to high chewing effort (e.g., meat, raw vegetables, and nuts).

### 2.2. Selection Criteria: Studies Were Selected Based on the Following Inclusion Criteria

Focus on aging populations (elderly, typically defined as individuals over 65 years of age).

Evaluation of masticatory function and its impact on oral and/or gastrointestinal health.

Investigation of the role of the oral microbiota in aging and its connection to gut health.

Clinical studies, randomized controlled trials (RCTs), and meta-analyses that address interventions to improve masticatory function or dietary intake in elderly individuals.

### 2.3. Exclusion Criteria Included Studies Focusing on Non-Human Subjects, Research That Did Not Directly Address Aging Populations, or Studies That Lacked Clear Methodological Reporting

Studies in which the primary population had neurological or musculoskeletal disorders severely impairing chewing function (e.g., Parkinson’s disease, post-stroke dysphagia) or was under medications known to affect mastication or digestion (e.g., anticholinergics, chemotherapy) were also excluded unless the chewing impact was specifically addressed and isolated.

### 2.4. Data Extraction

**Figure 1:**
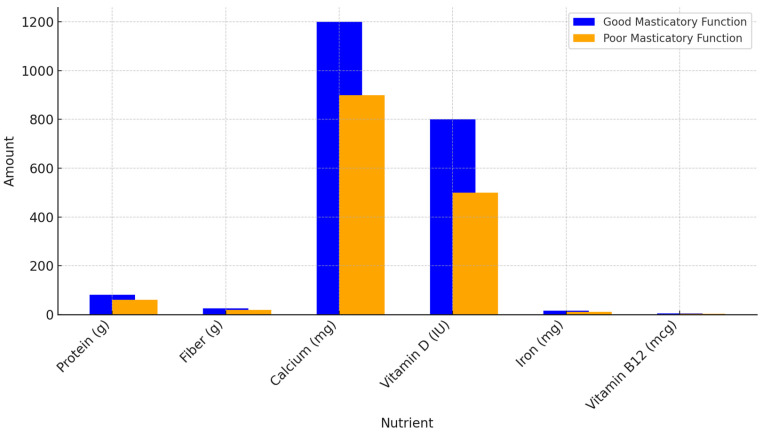
Histogram comparing nutrient intake between individuals with good and poor masticatory function. The bars represent the amount of each nutrient for both groups, illustrating how reduced masticatory function leads to lower nutrient intake in various categories. Data are reported as the mean intake (g or mg) of specific nutrients for individuals with good and poor masticatory function.

From each selected study, relevant data concerning the effect of impaired masticatory function on the oral microbiota, the gut microbiome, and nutritional outcomes were extracted. Particular attention was paid to outcomes such as changes in microbial diversity, gut inflammation, nutrient absorption, and clinical markers of malnutrition (e.g., body mass index, protein-energy malnutrition markers). The data presented in Figure 1 are based on representative averages extracted from previously published population studies, notably the Korean National Health and Nutrition Examination Survey (KNHANES) and Japanese cohorts of elderly individuals [1,17,18,19,20,21,22,23,24,25,26,27,28,29]. These studies assessed dietary intake in relation to masticatory capacity, classified using validated criteria, including the number of functional teeth, prosthetic use, and self-reported chewing difficulty.

### 2.5. Synthesis of Findings

A qualitative synthesis was performed, combining the results from experimental and clinical studies and clinical markers of malnutrition (e.g., body mass index, protein-energy malnutrition markers such as serum albumin, prealbumin, and transferrin levels, and total lymphocyte count) to draw conclusions about the interconnections between masticatory function, oral microbiota, and overall health of the elderly. Particular emphasis was placed on understanding how impaired mastication can lead to or exacerbate malnutrition and gastrointestinal disorders, and microbial imbalances in both the oral cavity and gut were assessed for their statistical significance using appropriate methods as reported by the original authors, typically including *t*-tests, ANOVA, chi-squared tests, and regression models depending on the data structure and study design.

### 2.6. Statistical Methods

While this review is primarily qualitative, studies that included quantitative data were assessed for their statistical significance using appropriate methods as reported by the original authors. Statistical analyses from selected studies were referenced to demonstrate the strength of the associations between masticatory function, oral microbiota changes, and nutritional outcomes.

### 2.7. Quality Assessment

The methodological quality of the studies included in this review was assessed based on established criteria for evaluating clinical research and systematic reviews. These included the risk of bias, sample size, and the robustness of the findings. Studies with higher methodological rigor were prioritized, and their conclusions were given more weight in the final synthesis.

### 2.8. Limitations

Limitations of the current review include the potential for publication bias, as studies with significant findings are more likely to be published. Additionally, while many studies have addressed the relationship between masticatory function and oral health, fewer have explored the detailed microbiological mechanisms that link the oral cavity and gut. Furthermore, the diversity of the interventions and outcomes across studies presented challenges in comparing results directly.

## 3. Results and Discussion

### 3.1. Impaired Masticatory Function and Nutritional Status

Numerous studies indicated that reduced masticatory function in older adults is strongly associated with inadequate nutritional intake, particularly in terms of essential nutrients such as protein, fiber, vitamins, and minerals. Elderly individuals with severe tooth loss or poor dentition often prefer soft, easy-to-chew foods, which are typically lower in nutritional value. For instance, protein intake, particularly from animal-based sources, is significantly reduced in individuals with impaired mastication, contributing to a higher risk of protein-energy malnutrition [1]. This dietary restriction can lead to deficiencies in micronutrients, including calcium and vitamin D, which are crucial for bone health and immune function [3,4,5].

### 3.2. Oral Microbiota and Gut Health

Several studies examined the impact of masticatory dysfunction on the oral microbiota and its potential link to gut health. Individuals with impaired chewing ability were found to have a reduced diversity of oral microbial species, with an overrepresentation of certain pro-inflammatory bacteria such as *Lactobacillus* and *Streptococcus* species [2,3,4,5,6]. This dysbiosis may contribute to local inflammation in the oral cavity and increase the risk of systemic inflammatory responses. Furthermore, these changes in the oral microbiota can be swallowed, influencing the gut microbiota and potentially contributing to gastrointestinal disturbances like constipation, altered bowel movements, and a higher risk of gut permeability and inflammation [7,8,9]. Chewing exercises may include the repetitive mastication of sugar-free gum, training with specially designed chewing aids or textured foods, and isometric exercises involving the jaw muscles. These activities aim to improve masticatory muscle tone, coordination, and bite force in elderly individuals with declining oral function [10]. Reduced mastication not only affects nutrient intake but also impairs digestive efficiency. Studies highlighted that elderly individuals with reduced masticatory function often experience slower gastric emptying and altered gastric acid secretion, both of which can impair nutrient digestion and absorption [3]. Additionally, impaired mastication can result in smaller food particles reaching the stomach, which hinders enzymatic activity and the absorption of vital nutrients. The oral cavity hosts a complex microbial ecosystem composed of over 700 bacterial species, along with viruses and fungi, structured into distinct niches such as the tongue, teeth, gingiva, and saliva. These microbial communities play a critical role in maintaining oral and systemic health, influencing processes such as immunity, nitric oxide metabolism, and the transmission of bacteria to the gut [5]. Dysbiosis in this ecosystem has been linked to periodontal disease, systemic inflammation, and even neurodegenerative conditions [6,7]. A lack of sufficient mastication also disrupts the natural balance of the gut microbiota by reducing the mechanical breakdown of food, which can alter fermentation processes and microbiota composition [10,11,12]. Interventions aimed at improving masticatory function, such as dental prosthetics, chewing exercises, and dietary modifications, were found to have positive effects on nutritional intake and overall health. For instance, elderly patients who received prosthetic dental treatments demonstrated an improvement in their ability to chew and, subsequently, in their nutrient intake. Studies also showed that older adults who received dietary counseling to include more high-fiber, nutrient-dense foods, despite having dental issues, could significantly improve their nutritional status and gut health [4,11,12,13,14]. Aging often leads to a decline in masticatory function, which can significantly impact nutrient intake. Several studies have shown that individuals with reduced chewing ability tend to consume fewer essential nutrients, including protein, fiber, vitamins, and minerals, which are vital for maintaining health. The histogram presented earlier in this manuscript compares nutrient intake between individuals with good and poor masticatory function. The bars represent the amount of each nutrient for both groups, illustrating how reduced masticatory function leads to lower nutrient intake in various categories. Several studies advocated for an integrated, multidisciplinary approach to the care of aging populations. The combination of oral health management and nutritional counseling was found to be particularly effective in promoting better health outcomes. Elderly individuals who received coordinated care from both nutritionists and dental professionals had better oral health, improved mastication, and were less likely to suffer from malnutrition [5]. This approach not only improves chewing function but also helps restore more-balanced oral and gut microbiota, thereby reducing inflammation and promoting better digestion [6,15,16,21,30].

The findings from the studies reviewed underscore the critical role of masticatory function in maintaining proper nutrition and gastrointestinal health in aging individuals. The deterioration of masticatory ability in older adults is not merely a localized oral issue but has far-reaching implications for overall health. As the elderly population continues to grow globally, the importance of preserving masticatory function through preventive care, rehabilitation, and dietary interventions cannot be overstated.

One of the most-consistent findings across studies is the link between impaired masticatory function and malnutrition in the elderly. As people age, dental health often deteriorates, which directly affects their ability to chew and subsequently impacts their dietary choices. This can lead to the insufficient intake of essential nutrients, including proteins, fiber, and micronutrients, putting older individuals at risk for protein-energy malnutrition and other nutrient deficiencies [21,30]. The interplay between poor mastication and nutrient intake forms a cycle that is difficult to break, as malnutrition itself can exacerbate masticatory dysfunction by reducing muscle strength, including the muscles involved in chewing [31]. On the left, the mouth shows the differences in bacterial diversity between individuals with good and poor masticatory function, while on the right, the intestine highlights how changes in the oral microbiota can influence the gut microbiota. Reduced masticatory function is associated with changes in microbial composition, potentially affecting both digestive and systemic health. Reduced mastication influences gut health not only by altering food texture and digestive enzyme activation but also by affecting gut microbiota composition. Poorly chewed food leads to larger food particles reaching the intestine, which impairs nutrient bioaccessibility and may modify fermentation patterns in the colon. Additionally, reduced cephalic-vagal stimulation decreases gastric acid and bile secretion, promoting dysbiosis. These changes have been associated with increased gut permeability, systemic inflammation, and symptoms such as constipation, bloating, and altered bowel habits [17,18,19].

The changes in the oral microbiota associated with impaired mastication are particularly concerning. Dysbiosis in the oral cavity can contribute to chronic low-grade inflammation, which is a hallmark of many age-related diseases, including cardiovascular disease, diabetes, and neurodegenerative conditions [17]. The oral microbiota is a primary source of microbes that are swallowed and can influence the gut microbiota, potentially leading to dysbiosis in the gastrointestinal tract as well. This bidirectional relationship between oral and gut microbiota underscores the importance of maintaining a healthy balance in both regions through proper oral hygiene and dietary interventions [18].

**Figure 2:**
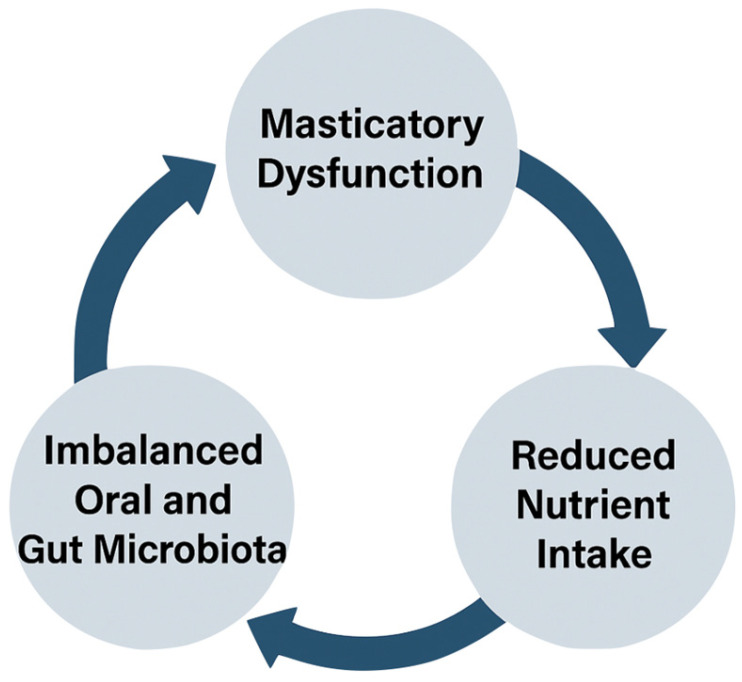
Linear diagram showing the vicious cycle linking poor masticatory function to malnutrition. Each stage flows into the next—from impaired chewing to reduced nutrient intake, leading to malnutrition and muscle loss. A dashed return arrow illustrates the feedback loop that perpetuates the cycle.

Gut health plays a central role in aging and overall well-being. Research has shown that the gut microbiota is involved in a variety of processes, from immune regulation to nutrient absorption and mental health [16,19,27,28,29,32,33]. Impaired mastication can disrupt these processes by influencing both the microbial balance in the mouth and the ability to digest food properly. Gut dysbiosis, often a result of poor diet and impaired mastication, is linked to a range of gastrointestinal disorders, including constipation, which is common in elderly populations [1,20,21,22,23,24,25,26]. Moreover, gut inflammation resulting from dysbiosis may contribute to systemic inflammation, further complicating the aging process. Each stage of the cycle is represented with simple icons: starting with poor masticatory function, leading to reduced nutrient intake, which fosters malnutrition and muscle loss, further worsening chewing ability [20,34]. This cycle contributes to the deterioration of nutritional and physical health in the elderly (Figure 2).

Given the impact of masticatory function on nutrition and health, interventions targeting chewing ability should be considered an integral part of nutritional care for elderly individuals. Dental rehabilitation, including the use of dentures or implants, along with masticatory exercises, can significantly improve chewing efficiency and, in turn, enhance dietary intake. Dietary counseling to encourage nutrient-dense foods, even in individuals with reduced chewing ability, is also crucial. Furthermore, integrating dental and nutritional care through a multidisciplinary approach can help optimize health outcomes by addressing both oral and nutritional issues. In addition to dental rehabilitation and dietary counseling, emerging strategies include targeted probiotic supplementation to rebalance oral and gut microbiota, biofeedback-based masticatory training, and the development of customized food textures for elderly individuals with dental impairments [35,36,37,38,39,40,41]. For example, studies from Japan and Northern Europe have demonstrated that interdisciplinary clinics—where dietitians and prosthodontists co-manage elderly patients—result in improved BMI, inflammatory markers, and reported quality of life. Furthermore, access to culturally appropriate nutrient-dense foods that are easy to chew may also play a crucial role, especially in institutionalized settings.

## 4. Conclusions

The evidence reviewed in this commentary highlights the critical importance of masticatory function, oral microbiota, and gut health in maintaining proper nutrition and overall well-being in aging individuals. As aging is inevitably associated with a decline in masticatory ability, it is essential to recognize the far-reaching consequences that this has on nutritional status, gut microbiota composition, and systemic health. Impaired mastication not only disrupts nutrient intake but also promotes oral dysbiosis, which can further exacerbate gastrointestinal and systemic inflammation, contributing to the progression of age-related diseases.

The findings underscore the need for a holistic, multidisciplinary approach to the care of older adults, which integrates both dental and nutritional management. Interventions aimed at improving masticatory function, such as dental rehabilitation and dietary counseling, can significantly improve nutrient intake, restore microbial balance, and reduce inflammation, thus enhancing quality of life and reducing the risk of malnutrition and frailty.

Future research should focus on better understanding the mechanistic pathways linking masticatory dysfunction to microbiota alterations and malnutrition, with an emphasis on developing targeted interventions that preserve or restore chewing ability and improve nutritional outcomes in the elderly. Given the increasing aging population globally, promoting oral and nutritional health through integrated care models is essential for supporting healthy aging and improving the overall quality of life for older individuals.
